# Risk Assessment Models for Heated Tobacco Products

**DOI:** 10.1111/risa.70317

**Published:** 2026-07-30

**Authors:** Lucia Maddalena, Beril Yildiz, Francesca Del Vecchio Blanco, Mario Rosario Guarracino

**Affiliations:** ^1^ Institute for High‐Performance Computing and Networking National Research Council Naples Italy; ^2^ InDatis s.r.l. Cassino Italy; ^3^ AOU Policlinico “Luigi Vanvitelli,” Naples Italy; ^4^ Department of Economics and Law University of Cassino and Southern Lazio Cassino Italy; ^5^ Laboratory of Algorithms and Technologies for Networks Analysis National Research University Higher School of Economics, Nizhny Novgorod Russia

**Keywords:** cancer risk, heated tobacco product, mathematical modeling, noncancer hazard, risk assessment

## Abstract

Heated tobacco products (HTPs) are marketed as alternatives to conventional cigarettes with a potential reduced risk profile. Yet, their actual impact on cancer and noncancer disease risk remains uncertain and requires rigorous quantitative assessment. In this study, we develop a unified and transparent computational framework for toxicological risk assessment of HTPs, integrating chemical emissions data with compound‐specific toxicological thresholds derived from regulatory agencies. Our work (i) systematically reviews and harmonizes existing risk models used in the literature, (ii) formulates generalizable mathematical models for estimating lifetime cancer risk, hazard quotients, and margins of exposure that account for population demographics, smoking habits, and compound characteristics, and (iii) validates these models by reproducing published results and exploring the sensitivity of risk estimates to model parameters and emission sources. Using emissions data from conventional cigarettes and HTPs, we quantify per‐compound and aggregated cancer and noncancer risks, and evaluate the relative risk reduction associated with switching from cigarettes to HTPs. The proposed risk analysis models provide a reproducible, extensible, and transparent approach for computational toxicology assessment, and can be readily applied to emerging nicotine and tobacco products within harm‐reduction evaluation paradigms.

## 1 Introduction

Heated tobacco products (HTPs) and electronic cigarettes (ECs) are generally considered to have a reduced toxicological profile compared to conventional cigarettes, based on aerosol chemical characterization. Nevertheless, this represents only one part of the overall assessment of HTPs and ECs as potential reduced‐risk alternatives to continued cigarette smoking. A reduction in harmful and potentially harmful constituents (HPHCs) relative to cigarette smoke does not necessarily translate into a proportional reduction of individual risk for users switching to these novel products, since this would imply that all HPHCs have equal toxicological potency. For this reason, health‐related claims such as “reduced exposure” or “reduced risk” require support from a weight‐of‐evidence approach grounded in a comprehensive scientific assessment, as highlighted in the seminal book by the Institute of Medicine (2001) and subsequent literature, for example, Hatsukami et al. (2005), Berman et al. (2015), Murphy et al. (2017), and Peitsch et al. (2018).

**FIGURE 1:**
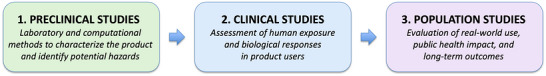
The three phases for risk assessment of novel tobacco products.

Following the draft guidance provided by the US FDA for evaluating Modified Risk Tobacco Products (MRTP), Murphy et al. (2017) propose a structured framework to assess the risk profile of novel tobacco products. The framework consists of three phases progressing from preclinical analyses to population‐level evaluation (see Figure 1), divided into 10 stages. Phase 1 encompasses preclinical studies starting with assessments of product design stability, followed by detailed chemical and physical characterization comparing HPHCs in novel products to those of reference cigarettes. This includes quantifying major aerosol constituents, performing untargeted and targeted analyses, and evaluating environmental emissions under standardized smoking regimens. In vitro regulatory toxicology then uses validated assays such as the Ames and Neutral Red Uptake tests to assess mutagenicity and cytotoxicity, employing both whole aerosol and aerosol condensates. At the core of the first phase is computational toxicology (Stage 4), which plays a pivotal role in translating chemical and in vitro findings into a quantitative understanding of risk. Using emissions data from previous stages, computational toxicology integrates exposure estimates with toxicological reference values to compute margins of exposure (EFSA Scientific Committee et al. 2025), identify toxicants of concern, and evaluate their relative contributions to both cancer and noncancer risk. This is complemented, when necessary, by mode‐of‐action (MOA) analyses and physiologically based pharmacokinetic (PBPK) modeling, which contextualize in vitro responses and help bridge biological effects across species, exposure scenarios, and dose ranges. Importantly, computational toxicology serves as the first point at which different streams of preclinical evidence converge quantitatively, and therefore, it provides the analytical foundation upon which later phases build. Phase 1 concludes with disease‐relevant in vitro models and systems science studies that investigate perturbations in biological pathways tied to oxidative stress, cardiovascular disease, chronic obstructive pulmonary disease, cancer, and inflammation using transcriptomic and network‐based analyses. Building on these preclinical insights, Phase 2 comprises clinical studies designed to validate whether differences observed in aerosol chemistry and computationally estimated toxicological risks translate into measurable changes in users. Because the clinical interpretation of biomarkers of exposure and biomarkers of biological effect relies critically on expectations formed in Stage 4, computational toxicology acts as the analytical bridge between exposure reductions and anticipated clinical responses. Puffing behavior, consumption patterns, pharmacokinetics, abuse liability, and omics‐based biomarker assessments are interpreted within the risk landscape shaped by the computational models. Finally, Phase 3 focuses on population studies evaluating risk perception, uptake behavior, marketing influences, and long‐term health outcomes. Postmarket surveillance and epidemiological modeling aim to detect unintended effects and estimate population‐level health impacts, particularly in the absence of long‐term epidemiological data.

Given the central role of computational toxicology in quantitatively linking chemical characterization, toxicological potency, exposure patterns, and expected biological outcomes, this paper focuses on preclinical risk assessment, emphasizing how computational modeling can be used to derive cancer and noncancer risk indicators for HTPs in a transparent and systematically validated manner. The aims of this study are: (a) a computational model for correlating toxicological data with the relative risk estimate associated with HTPs compared to traditional cigarettes validated on machine‐generated emission data; (b) a collection of existing toxicological data freely available in the literature; and (c) a replicable and transparent risk estimation methodology, also applicable to other harm reduction products.

## 2 Literature on Risk Assessment Models

Here, we summarize papers relevant to our research, sorted alphabetically by the surname of the first author. We start with reviews covering risk assessment, and then focus on three scientific papers taken as examples of risk assessment models that can be found in the literature; further scientific papers covering alternative models are provided in Appendix A.

### 2.1 Reviews

Cordery et al. (2024) extended their previous review Malt et al. (2022), including papers till 2023. Descriptions and main results were organized into several groups: (1) aerosol chemistry, (2) in vitro toxicology, (3) in vivo toxicology, (4) biomarkers, (5) nicotine pharmacokinetics and abuse liability, (6) health effects, and (7) HTPs, indoor air quality, and bystander exposure. Concerning toxicological risk exposure and health/cancer risk (CR) assessment (included in the first group), they reviewed Esposito et al. (2022), Lu et al. (2022), and Kusonić et al. (2023). They concluded that the available evidence indicates that HTP use may be associated with reduced cancer‐ and non‐cancer‐related risk when compared to cigarette smoking, even though further research is needed, and additional epidemiological studies into the long‐term effects of HTP use may help to determine the absolute CR associated with HTP use.

In Dempsey et al. (2023), the authors described an approach for *preliminary toxicological assessment* of HTPs that could be seen as a standard approach for future emerging products in this category. This phase of assessment is different from the reduced risk assessment, and it aims at assessing some basic standards to which all products should be held. For example, for HTPs, this might involve establishing that no combustion occurs during the use of the product, there is an overall reduction in HPHCs, there is some indication of reduced toxicity in in vitro systems, and all components and ingredients of both consumables and devices are appropriate for intended use and will not present new or increased toxicity to the final product. To this end, the authors reviewed the published literature on related studies performed on all kinds of HTPs, outlined a proposed approach that is consistent with regulatory requirements, and provided a logical approach to the preliminary toxicological assessment of HTPs.

Ghazi et al. (2024) is a scoping review of the toxicity and health impact of a specific HTP, namely, the Tobacco Heating System (THS) by Philip Morris International (PMI). Publications were categorized into two general categories: (1) toxicity assessments, including in vitro, in vivo, and systems toxicology studies; and (2) impact on human health, including clinical studies assessing biomarkers of exposure and biomarkers of health effects. Few studies were considered related to the estimation of cancer and noncancer risks (Rodrigo et al. 2021; Lachenmeier et al. 2018), and one related to the case of second‐hand smoke (Hirano and Takei 2020).

Mallock et al. (2019) provided a summary of assessments on HTPs and related challenges, grouping them into emissions and risk assessment, highlighting that the reliability and reproducibility of emission data are crucial factors for a subsequent risk assessment. Reviewed risk assessment approaches include Fowles and Dybing (2003), Stephens (2018), and Lachenmeier et al. (2018).

Malt et al. (2022) presented a narrative review on HTPs, that extends and updates previous reviews (Jankowski et al. 2019; Mallock et al. 2019; Simonavicius et al. 2019; Ratajczak et al. 2020) as well as smaller reviews (Basaran et al. 2019; Signes‐Costa et al. 2019; Znyk et al. 2021), and three journal issues published by PMI and British American Tobacco Investments Ltd. relating to their HTPs. The section on toxicological risk assessment modeling reviewed Stephens (2018), Lachenmeier et al. (2018), Slob et al. (2020), and Rodrigo et al. (2021), concluding that further research is required regarding the comparison between relative and absolute risk associated with HTP use, as well as differences between changes in single compounds and changes in cumulative levels.

### 2.2 Scientific Papers

#### 2.2.1 Models by Kusoni $\;\overset{´}{c}$ et al. (2023)

Kusonić et al. (2023) compared the noncarcinogenic and carcinogenic risk of HTP products and conventional cigarettes, computing the margin of exposure (MOE) and the Excess Lifetime Cancer Risk (ELCR) using the formula obtained from the official method proposal of the National Institute of Public Health and the Environment of the Netherlands and the EPA Guidelines, respectively. Specifically, for each compound i, MOE is computed as

(1) $$\begin{array}{c} MOE_{i}=\frac{PoD_{i}}{(Dabs_{i}/BW)*NCd*TransferRate}, \end{array}$$

where

- $PoD_{i}$ (mg/kg BodyWeight/day) indicates the toxicological Points of Departure (PoD) for compound i, being either NOAEL (No Observed Adverse Effect Level), LOAEL (Lowest Observed Adverse Effect Level), or BMDL (BenchMark Dose Level);
- $Dabs_{i}$ (mg/item) indicates the absorbed Dose of the compound i, estimated by $Dabs_{i}$ = 0.7*$Dcig_{i}$, $Dcig_{i}$ (in mg/item) being the amount of the compound i in the aerosol or mainstream smoke;
- BW (kg) indicates the human Body Weight, set to the default value of 70 kg;
- NCd (item/day) is the Number of Cigarettes smoked per day (in the experiments varying as 1, 5, 10, 15, 20, 25, 30, 35, and 40);
- TransferRate (%) of the product component into aerosol or smoke; if unknown, it is taken as 100%.

The cutoff point for the public health safety is set to MOE = 100 for noncarcinogenic analytes, while for the analytes classified by IARC (2026) as carcinogenic (IARC classes 1, 2A, or 2B), it is set to 1000 if the values for PoD originate from human studies and 10,000 if they originate from animal studies. MOE was not computed for compounds lacking PoD information.

The ELCR for a carcinogenic analyte i was computed according to the EPA Guidelines for Carcinogen Risk Assessment as

(2) $$\begin{array}{c} ELCR_{i}=Dcig_{i}*\frac{NCd}{BW}*SF_{i}, \end{array}$$

where $SF_{i}$ ((mg/kg BodyWeight/day)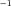) indicates the cancer Slope Factor for compound i. An ELCR lower than 1 in 1,000,000 ($10^{-6}$) is treated as essentially negligible, while an acceptable ELCR should not exceed $10^{-4}$. The ELCR was not computed for analytes lacking the SF value.

**FIGURE 2:**
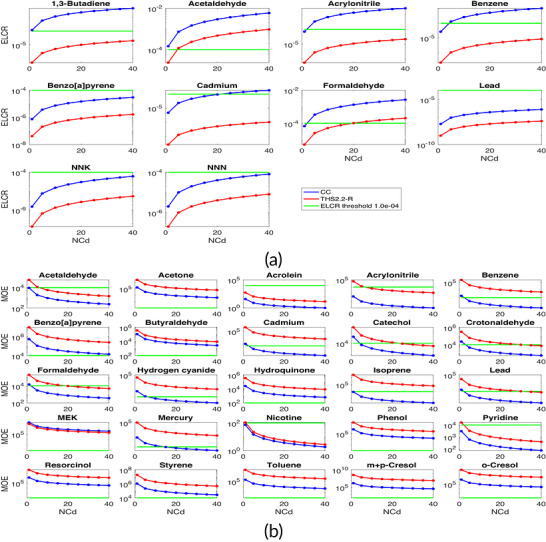
Results obtained using the (a) ELCR and (b) MOE models from Kusonić et al. (2023) on data by Jaccard et al. (2017) for the THS‐2.2‐R and CCs, varying the number of sticks/cigarettes smoked per day.

Available parameter values for the model include: IARC classification, experimental animal, toxicological thresholds (NOAEL, LOAEL, or BMDL, and related $PoD_{i}$ values), value and reference for cancer Slope Factors $SF_{i}$ used for the computation of $MOE_{i}$ and $ELCR_{i}$ of the chosen HPHCs present in conventional cigarette smoke and HTP aerosol (their Supplementary Table 1). Although not clearly stated, experiments were carried out on emission data obtained under the Health Canada Intense (HCI) smoking regimen by Jaccard et al. (2017) for THS2.2 with regular sticks (THS2.2‐R) and by Counts et al. (2005) for commercial cigarettes (CCs). Their results could be replicated, as shown in Figure 2 for ELCR and MOE.

**TABLE 1:** LCR results for 73 compounds computed using the model in Equation (17) on data for the 3R4F reference cigarette University of Kentucky (2008) and the THS2.2‐R, and percentage reduction in using the HTP compared to the cigarette.

Compound	CAS	LCR	LCR	LCR
		3R4F	THS2.2‐R	% Red.
2‐Aminonaphthalene	91‐59‐8	7.49e−06	1.31e−08	99.83
Cadmium	7440‐43‐9	4.42e−04	8.26e−07	99.81
1‐Aminonaphthalene	134‐32‐7	1.10e−05	2.60e−08	99.76
Isoprene	78‐79‐5	4.76e−03	1.13e−05	99.76
1,3‐Butadiene	106‐99‐0	1.50e−02	4.46e−05	99.70
Acrylonitrile	107‐13‐1	8.43e−03	4.66e−05	99.45
A‐alpha‐C	26,148‐68‐5	2.14e−05	1.21e−07	99.43
4‐Aminobiphenyl	92‐67‐1	1.76e−05	1.25e−07	99.29
Benzene	71‐43‐2	2.61e−03	1.85e−05	99.29
Ethylene oxide	75‐21‐8	1.16e−01	1.01e−03	99.13
NPYR	930‐55‐2	1.33e−05	1.21e−07	99.09
Ethylbenzene	100‐41‐4	3.71e−05	3.38e−07	99.09
Naphthalene	91‐20‐3	2.62e−05	2.61e−07	99.00
Me‐A‐alpha‐C	68,006‐83‐7	6.64e−06	7.35e−08	98.89
5‐Methylchrysene	3697‐24‐3	3.43e−06	4.62e−08	98.65
Trp‐P‐2	62,450‐07‐1	5.85e−06	1.03e−07	98.24
NPIP	100‐75‐4	1.21e−05	2.32e−07	98.08
Trp‐P‐1	62,450‐06‐0	3.43e−05	7.25e−07	97.88
Vinyl chloride	75‐01‐4	7.55e−06	1.72e−07	97.72
Quinoline	91‐22‐5	3.74e−04	1.12e−05	97.01
NNK	64,091‐91‐4	3.64e−03	1.12e−04	96.93
o‐Anisidine	90‐04‐0	1.87e−07	6.01e−09	96.78
Beryllium	7440‐41‐7	1.41e−06	4.67e−08	96.70
Octa CDD	3268‐87‐9	2.20e−06*	7.92e−08*	96.40
NNN	16,543‐55‐8	1.11e−04	4.64e−06	95.84
NEMA	10,595‐95‐6	3.67e−05	1.60e−06	95.64
Indeno[1,2,3‐cd]pyrene	193‐39‐5	4.93e−07	2.76e−08	94.40
Benzo[a]pyrene	50‐32‐8	1.52e−05	8.81e−07	94.21
Benzo[b]fluoranthene	205‐99‐2	1.21e−06	7.77e−08	93.57
Benzo[a]anthracene	56‐55‐3	2.85e−06	1.85e−07	93.50
NDEA	55‐18‐5	1.83e−04	1.32e−05	92.78
Chrysene	218‐01‐9	3.14e−07	2.31e−08	92.64
IQ	76,180‐96‐6	2.95e−06	2.33e−07	92.10
Dibenzo[a,h]anthracene	53‐70‐3	1.41e−06	1.39e−07	90.19
Formaldehyde	50‐00‐0	9.78e−04	9.92e−05	89.86
NDMA	62‐75‐9	1.53e−04	1.62e−05	89.42
Arsenic	7440‐38‐2	3.44e−05	4.14e−06	87.96
Propylene oxide	75‐56‐9	4.29e−06	5.17e−07	87.95
Acetaldehyde	75‐07‐0	4.49e−03	5.55e−04	87.64
Dibenzo[a,i]pyrene	189‐55‐9	1.16e−05	1.45e−06	87.45
Dibenzo[a,e]pyrene	192‐65‐4	7.47e−07	9.39e−08	87.44
Benzo[k]fluoranthene	207‐08‐9	3.35e−07	4.28e−08	87.24
Hydrazine	302‐01‐2	4.98e−05	1.00e−05	79.94
Lead	7439‐92‐1	3.48e−07	7.30e−08	79.01
Acetamide	60‐35‐5	2.61e−04	7.05e‐05	72.97
Ethyl carbamate	51‐79‐6	1.86e−06	5.60e−07	69.98
Nitrobenzene	98‐95‐3	3.39e−06	1.03e−06	69.51
Acrylamide	79‐06‐1	5.87e−03	2.21e−03	62.38
Glu‐P‐1	67,730‐11‐4	3.35e−07	1.33e−07	60.25
Glu‐P‐2	67,730‐10‐3	1.20e−07	4.80e−08	60.13
Dibenzo[a,h]pyrene	189‐64‐0	3.61e−06	1.55e−06	57.03
NMOR	59‐89‐2	1.05e−06	5.23e−07	50.00
NDELA	1116‐54‐7	1.67e−07	8.36e−08	49.94
Dibenzo[a,l]pyrene	191‐30‐0	3.41e−06	1.88e−06	45.02
Chromium	7440‐47‐3	1.31e−03	7.71e−04	41.20
Nickel	7440‐02‐0	4.55e−06	3.00e−06	33.99
2,3,7,8‐Tetra CDF	51,207‐31‐9	1.44e−05*	1.10e−05*	23.68
2,3,4,7,8‐Penta CDF	57,117‐31‐4	4.40e−05*	3.85e−05*	12.50
1,2,3,7,8‐Penta CDD	40,321‐76‐4	1.52e−04*	1.33e−04*	12.50
1,2,3,7,8‐Penta CDF	57,117‐41‐6	4.18e−06*	3.85e−06*	7.89
2,3,7,8‐Tetra CDD	1746‐01‐6	1.48e−04*	1.41e−04*	5.13
1,2,3,4,7,8‐Hexa CDD	39,227‐28‐6	1.41e−05*	1.41e−05*	0.00
1,2,3,6,7,8‐Hexa CDD	57,653‐85‐7	1.41e−05*	1.44e−05*	−2.70
1,2,3,7,8,9‐Hexa CDD	19,408‐74‐3	1.25e−05*	1.29e−05*	−3.03
1,2,3,4,6,7,8‐Hepta CDF	67,562‐39‐4	9.50e−07*	1.03e−06*	−8.00
2,3,4,6,7,8‐Hexa CDF	60,851‐34‐5	9.12e−06*	9.88e−06*	−8.33
Cobalt	7440‐48‐4	1.50e−05	1.62e−05	−8.59
1,2,3,4,7,8,9‐Hepta CDF	55,673‐89‐7	1.25e−06*	1.37e−06*	−9.09
1,2,3,4,7,8‐Hexa CDF	70,648‐26‐9	8.36e−06*	9.12e−06*	−9.09
1,2,3,6,7,8‐Hexa CDF	57,117‐44‐9	7.98e−06*	8.74e−06*	−9.52
1,2,3,7,8,9‐Hexa CDF	72,918‐21‐9	9.88e−06*	1.10e−05*	−11.54
Octa CDF	39,001‐02‐0	4.84e−08*	5.94e−08*	−22.73
o‐Toluidine	95‐53‐4	5.10e−06	1.20e−05	−135.23

*LCR results for compounds whose means come from a single data source.

#### 2.2.2 Models by Pack et al. (2018)

Pack et al. (2018) estimated the risk associated with the exposure to 38 hazardous constituents in the mainstream smoke of five low‐yield cigarettes sold in Korea. Risk calculations were performed using algorithms from previous studies and Korean population‐based exposure parameters under both the ISO3308:2012 (2012) and ISO20778:2018 (2018) regimens, in the following named ISO and HCI for brevity, respectively. The authors adopt a probabilistic approach using Monte Carlo simulations, where the risk for each compound is represented as a probability distribution to account for uncertainty arising from variations in exposure parameters. The equations for risk computation are as follows.

The calculated dose $CD_{i}$ of constituent i of cigarette smoke via inhalation (µg/cig) is

(3) $$\begin{array}{c} CD_{i}=CY_{i}*\frac{RR_{i}}{100}*\frac{100-MS}{100}, \end{array}$$

with $CY_{i}$ (µg/cig) mainstream smoke Yield for Constituent i, $RR_{i}$ (%) Respiratory Retention rate for constituent i, and MS (%) Mouth‐Spill rate.

The Average Daily Concentration $EC_{i}$ of constituent i (µg/m^3^) is

(4) $$\begin{array}{c} EC_{i}=\frac{CD_{i}*NCd}{IR}, \end{array}$$

with NCd (cig/day) average Number of Cigarettes smoked per day and IR (m^3^/day) daily Inhalation Rate.

The Hazard Quotient $HQ_{i}$, which represents the potential of noncancer risk of constituent i, is then computed as

(5) $$\begin{array}{c} HQ_{i}=\frac{EC_{i}}{RfC_{i}}, \end{array}$$

with $RfC_{i}$ (µg/m^3^) Reference Concentration for the inhalation route of the exposure of the *i*th constituent. HQ was used to evaluate the potential of noncarcinogenic adverse health effects of 31 constituents. If the $HQ_{i}$ value was greater than 1 (i.e., the estimated average daily concentration in humans $EC_{i}$ exceeded the reference concentration $RfC_{i}$), it was considered that there was a potential health risk. On the other hand, if $HQ_{i}$ was less than 1, it was concluded that there was no potential for adverse health effects due to the target compounds.

The Incremental Lifetime Cancer Risk $ILCR_{i}$ of carcinogen i is computed as

(6) $$\begin{array}{c} ILCR_{i}=EC_{i}^{life}*IUR_{i}, \end{array}$$

where $IUR_{i}$ is the IUR of carcinogen i, expressed in (µ/m^3^)^−1^ (Stephens 2018), and $EC_{i}^{life}$ (µg/m^3^) is the Lifetime Average Daily Concentration of the *i*th constituent

(7) $$\begin{array}{c} EC_{i}^{life}=\frac{CD_{i}*NCy*\left(LE-SA\right)}{IR*LE*365}, \end{array}$$

with NCy (cig/year) average Number of Cigarettes smoked per year, LE Life Expectancy, and SA Age at Smoking initiation. ILCR was calculated to estimate the CR of 18 carcinogens. The EPA has set a target range of $10^{-6}$ to $10^{-4}$ to manage CR, and, generally, over $10^{-4}$ of CR is considered unacceptable.

**FIGURE 3:**
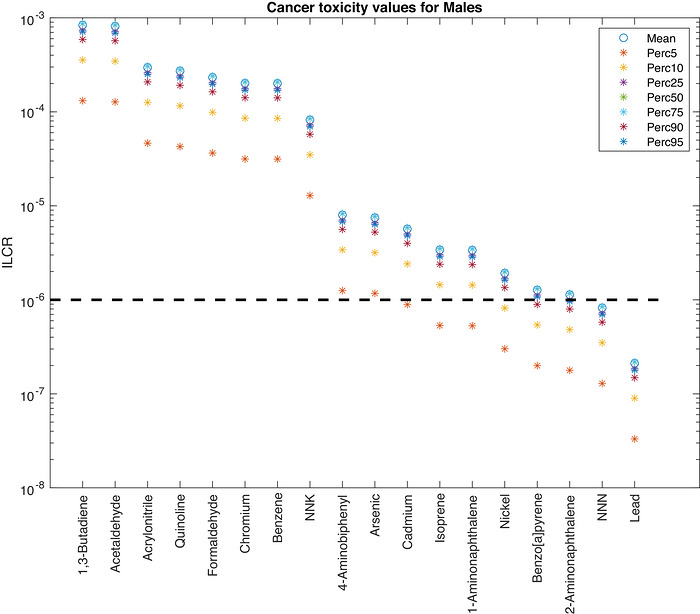
ILCR results obtained using the model from Pack et al. (2018) on their data for 18 compounds in CC smoke under the HCI smoking regimen with parameter values for males.

Available data include mean and standard deviation (over the five CCs, three replicates each) of the mainstream smoke yields $CY_{i}$ for 38 compounds under ISO and HCI regimens (their Table 3). Parameter values for risk assessment include values for $RR_{i}$, $RfC_{i}$, and $IUR_{i}$ for the 38 compounds (their Table S2), as well as descriptive statistics (means and percentiles) of exposure parameters (MS,IR,NCd, NCy,LE,SA, also distinguished by sex) for cancer and noncancer risk calculation (their Table S3). Their ILCR results for 18 constituents (divided by sex) from 10,000 Monte Carlo simulations are shown as box plots (their Figure 1). Their HQ results for 31 constituents (divided by sex) using 10,000 Monte Carlo simulations are also provided as mean, median, 5th and 95th percentiles (their Table 4). We computed ILCR values for the fixed values of exposure parameters from their Table S3 (i.e., we did not obtain 10,000 values varying these parameters), obtaining the results shown in Figure 3, which agree with their results.

We could also use the same formulas to compute the ILCR for any product and any concentration data file. However, even though $IUR_{i}$ and $RfC_{i}$ values for several other compounds could be obtained by online resources, information on $RR_{i}$ could be found only for very few other compounds (e.g., Lu et al. 2022); this is needed for computing $CD_{i}$ in Equation (3) and thus for both $HQ_{i}$ and $ILCR_{i}$.

#### 2.2.3 Models by Rodrigo et al. (2021)

Rodrigo et al. (2021) performed risk assessment for HPHCs from eight commercial HTPs and two ECs when compared with those from 273 CCs. They adopted cancer potency values (computed as in Stephens (2018), with slight modifications) and MOE (computed as in Baumung et al. 2016; Lachenmeier et al. 2018) for quantifying cancer and noncancer risks, respectively, associated with exposure to HPHCs from the considered HTPs when compared with those from CCs on the basis of available compound‐specific toxicological threshold references from official regulatory agencies.

For each product type P (HTP, EC, or cigarette), they computed the Cancer Potency $CP^{P}$ of the product as the sum of the n compound concentrations $C_{i}^{P}$ weighted by their $IUR_{i}$ (published by OEHHA 2026 or EPA 2026)

(8) $$\begin{array}{c} CP^{P}=\sum_{i=1}^{n}IUR_{i}*C_{i}^{P}, \end{array}$$

where $IUR_{i}$ are expressed in (µg/m^3^)^−1^, while $C_{i}^{P}$ are expressed in µg/100 mL. They estimate the Lifetime Cancer Risk $LCR^{P}$ for product P as

(9) $$\begin{array}{c} LCR^{P}=\frac{DAI}{DBV}*CP^{P}, \end{array}$$

where DBV is the Daily Breathed Volume (in m^3^, set to 20 for all products) and the Daily Aerosol Intake DAI (in mL) is computed as

(10) $$\begin{array}{c} DAI=\left\{\begin{array}{cc} PuffV*PuffN*DC & \mathrm{for}\mathrm{stick}-\mathrm{based}\mathrm{HTPs}\mathrm{and}\mathrm{cigarettes}\\ 0.001*DC & \mathrm{for}\mathrm{liquid}-\mathrm{based}\mathrm{HTPs}\mathrm{and}\mathrm{ECs}, \end{array}\right. \end{array}$$

where the puff volume PuffV is set to 55 mL, the number of puffs PuffN varies with the product, and DC is the Daily Consumption, set to 20 cigarettes/sticks or 20 L.

For each product P and each compound i, the MOE is computed as

(11) $$\begin{array}{c} MOE_{i}^{P}=\frac{IEL_{i}*DBV}{DAI*C_{i}^{P}}, \end{array}$$

where the Inhalation Exposure Limit $IEL_{i}$ of compound i (in mg/m^3^) is the lowest among publicly available toxicological thresholds:

(12) $$\begin{array}{c} IEL_{i}=\min\left\{DNEL_{i},REL_{i},RfC_{i}\right\}, \end{array}$$

with Derived No Effect Level $DNEL_{i}$ as set by EChA (2026), Reference Exposure Level $REL_{i}$ as set by OEHHA, and Reference Concentration $RfC_{i}$ as set by EPA.

The combined MOE for each product, $MOE_{T}^{P}$, that can be seen as a rough estimate of additive cumulative exposure (Lachenmeier et al. 2018), computed as

(13) $$\begin{array}{c} MOE_{T}^{P}=\frac{1}{\sum_{i=1}^{n}\frac{1}{MOE_{i}^{P}}} \end{array}$$

is evaluated both including and excluding the MOE for nicotine.

Available data for chemical characterization include the concentration $C_{i}$ of 43 HPHC yields in the aerosol from the eight evaluated HTP brands (µg/100 mL) acquired under the HCI smoking regimen (their Suppl. Table 5). The unspecified association of these values to the specific products and puff number PuffN (their Suppl. Table 4) can be inferred only by a detailed analysis of possible combinations. For the two ECs and the CCs, only minimum and maximum values are reported (their Table 3), based on data from Nicol et al. (2020). Parameter values for risk assessment include: (a) the list of 21 compounds considered for computing CP, with their IARC type, the $IUR_{i}$, and the $IUR_{i}$ origin (their Table 1), and (b) the list of 35 compounds considered for computing their MOE (13 in common with the previous group), with their $IEL_{i}$ (mg/m^3^) and IEL origin (their Table 2).

## 3 The CR Model

To improve the relevance for risk assessment, we take into account the smoke/aerosol spilled from the mouth before inhalation (Mouth‐Spill [MS]) and the Respiratory Retention (RR) during the inhalation cycle, by transforming the exposure estimates of the compounds into calculated doses. Following St. Charles et al. (2013), the calculated dose $CD_{i}$ of compound i in the cigarette smoke/HTP aerosol via inhalation (µg/item) is set to

(14) $$\begin{array}{c} CD_{i}=C_{i}*\frac{RR_{i}}{100}*\left(1-\frac{MS}{100}\right), \end{array}$$

with

- $C_{i}$ (µg/item) yield for compound i in the mainstream smoke/aerosol;
- $RR_{i}$ (%) Respiratory Retention rate for compound i, that is, the portion of the compound in the smoke/aerosol inhaled and retained in the respiratory tract;
- MS (%) Mouth‐Spill rate, that is, the portion of the smoke/aerosol that leaks out from the smoker's mouth when the item (cigarette/stick) is removed from the mouth.

The daily Exposure Concentration $EC_{i}$ of compound i (µg/m^3^) is given as

(15) $$\begin{array}{c} EC_{i}=\frac{CD_{i}*NCd}{DBV}, \end{array}$$

with

- NCd (item/day) average number of cigarettes/sticks smoked per day;
- DBV (m^3^/day) daily breathed volume (often called inhalation rate).

The Lifetime Exposure Concentration $LEC_{i}$ of compound i (µg/m^3^) is given as

(16) $$\begin{array}{c} LEC_{i}=EC_{i}*\frac{NCy}{NCd*365}*\frac{LE-SA}{LE}, \end{array}$$

with

- NCy (item/year) average number of cigarettes smoked per year;
- LE (years) life expectancy;
- SA (years) age at smoking initiation.

The Lifetime Cancer Risk $LCR_{i}$ of a carcinogenic compound i is computed as

(17) $$\begin{array}{c} LCR_{i}=LEC_{i}*IUR_{i}, \end{array}$$

where $IUR_{i}$ (µg/m^3^)^−1^ is the Inhalation Unit Risk of carcinogen i, that is, the lifetime cancer risk from continuous inhalation exposure to 1 µg of the carcinogen per m^3^ of air. As generally agreed, an LCR lower than 1 in 1,000,000 ($10^{-6}$) is treated as essentially negligible, while an acceptable LCR should not exceed $10^{-4}$.

The model in Equation (17) is general enough to exploit any available information on population demographics (via LE), smoke habits (via SA, MS, NCd, NCy, and DBV), and compound characteristics (via $RR_{i}$ and $IUR_{i}$). CR assessment models adopted by other authors can be proven to be specific cases of the described model, taking into account‐specific hypotheses and available information. Indeed,

- in the frequent case of models disregarding MS and RR (e.g., Kusonić et al. 2023; Rodrigo et al. 2021), the calculated dose $CD_{i}$ of Equation (14) coincides with the compound yield $C_{i}$, as it is assumed no MS (MS=0) and full RR ($RR_{i}$=100);
- under the usual assumption that NCy=NCd∗365, the first fraction in Equation (16) is equal to 1; the second fraction indicates the effective exposure duration (generally denoted as ED) over the life expectancy, often assumed as averaging time (generally denoted as AT) for assessing carcinogens (e.g., Marano et al. 2018; Hirn et al. 2020);
- in the case of models adopting the Slope Factors $SF_{i}$ rather than the $IUR_{i}$ (e.g., Kusonić et al. 2023; Lu et al. 2022), Equation (17) can still be computed considering that
(18) $$\begin{array}{c} SF_{i}=\frac{BW}{DBV}*IUR_{i}*10^{3}, \end{array}$$
  where $SF_{i}$ is expressed in (mg/kg of body weight per day)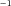 and BW is the body weight (kg).

Table 1 compares the LCR results for 73 compounds found in the mainstream smoke of the 3R4F reference cigarette with those for the same compounds found in the aerosol of the THS2.2‐R under the HCI smoking regimen, and provides the LCR percentage reduction in using the HTP compared to the 3R4F cigarette, that is,

(19) $$\begin{array}{c} LCR_{i}\;\%\;Reduction=100*\left(1-\frac{LCR_{i}^{HTP}}{LCR_{i}^{3R4F}}\right). \end{array}$$

Specifically, emission data ($C_{i}$ in Equation 14) are the pooled means (i.e., means weighted by the number of replicates) of measures included in the HTP‐AeroChem data set (Maddalena et al. 2026) for the two products, coming from up to 28 different sources. The parameters for the LCR model were chosen as follows: (a) $RR_{i}=100$ and MS=0 in Equation (14), thus disregarding RR rates and MS; (b) NCd = 20 and DBV = 20 in Equation (15); (c) NCy=365∗NCd, LE = 70, and SA = 0 in Equation (16); and (d) the $IUR_{i}$ in Equation (17) are the most conservative among the available values, that is,

(20) $$\begin{array}{c} IUR_{i}=\max\left\{IUR_{i}^{EPA},\;IUR_{i}^{OEHHA},\;IUR_{i}^{H},\;IUR_{i}^{L},\;IUR_{i}^{M},\;IUR_{i}^{P},\;IUR_{i}^{R}\right\}, \end{array}$$

where $IUR_{i}^{EPA}$ and $IUR_{i}^{OEHHA}$ are the IURs provided by EPA and OEHHA, and $IUR_{i}^{H}$, $IUR_{i}^{L}$, $IUR_{i}^{M}$, $IUR_{i}^{P}$, and $IUR_{i}^{R}$ indicate those reported in Hoshino et al. (2025), Lu et al. (2022), Marano et al. (2018), Pack et al. (2018), and Rodrigo et al. (2021), respectively (see the Supporting Information). These choices for the emission data and for parameter values allowed the model in Equation (17) to provide the most comprehensive set of LCR results that can be found in the literature. In Table 1, we can observe that 11 compounds in the 3R4F smoke and 34 in the HTP aerosol have negligible LCR ($<10^{-6}$), while 18 compounds in the 3R4F smoke and seven in the HTP aerosol are unacceptable for CR ($LCR>10^{-4}$). Moreover, the LCR for 61 (out of 73) compounds included in the HTP aerosol is reduced compared to the same compounds in the 3R4F cigarette smoke by a factor between 5.13% and 99.83%, depending on the compound. Finally, it appears that for 12 compounds the LCR for the 3R4F cigarette remains unchanged or increases compared to the LCR for the HTP; this is mainly due to the fact that the emissions for these compounds, coming from a single data source, generated values below the Limit of Detection (LOD) and were thus imputed as the LOD value itself. Indeed, only for two compounds (Cobalt and o‐Toluidine) the LCR increases according to two or more data sources.

## 4 The Noncancer Hazard Models

The effects of smoke/aerosol constituents for diseases other than cancer are generally estimated using the HQ or the MOE. The detailed models adopted for our experiments are described below.

### 4.1 Hazard Quotient

The Hazard Quotient $HQ_{i}$ of compound i is the ratio of the potential exposure to the compound and the level at which no adverse effects, other than cancer, are expected. Here, it is computed as

(21) $$\begin{array}{c} HQ_{i}=10^{-3}\frac{EC_{i}}{RfC_{i}}, \end{array}$$

where $EC_{i}$ (µg/m^3^) is the daily Exposure Concentration given in Eq. (15) and $RfC_{i}$ (mg/m^3^) is the Reference Concentration for the inhalation route of the exposure of the *i*‐th compound.

Similarly to the CR model described in Section 3, the formulation of the noncancer hazard model of Equation (21) is general enough to exploit any available information on smoke habits (via MS, NCd, and DBV) and compound characteristics (via $RR_{i}$ and $RfC_{i}$). The higher the HQ, the greater the health hazard of the compound. If the HQ_*i*_ value is greater than 1 (i.e., the estimated average daily concentration $EC_{i}$ exceeds the reference concentration $RfC_{i}$), it is considered that there is a potential health risk. On the other hand, if the HQ_*i*_ is less than 1, it can be concluded that there is no potential for adverse health effects due to compound i.

Table 2 compares the HQ results for 53 compounds found in the mainstream smoke of the 3R4F reference cigarette with those for the same compounds found in the aerosol of the THS2.2‐R under the HCI smoking regimen, and provides the HQ percentage reduction in using the HTP compared to the 3R4F cigarette, that is,

(22) $$\begin{array}{c} HQ_{i}\;\%\;Reduction=100*\left(1-\frac{HQ_{i}^{HTP}}{HQ_{i}^{3R4F}}\right). \end{array}$$

Specifically, the emission data and model parameter values are the same as those adopted for the comparisons in Table 1, while values for $RfC_{i}$ in Equation (21) are the most conservative among the available values, that is, the minima in the set

(23) $$\begin{array}{c} \{DNEL_{i},\;REL_{i},\;RfC_{i}^{EPA},\;RfC_{i}^{PPRTV},\;RfC_{i}^{H},\;RfC_{i}^{L},\;RfC_{i}^{M},\;RfC_{i}^{P},\;RfC_{i}^{R}\}, \end{array}$$

where the $DNEL_{i}$ is set by EChA, the $REL_{i}$ is set by OEHHA, the Reference Concentration $RfC_{i}^{EPA}$ is set by EPA, the Reference Concentration $RfC_{i}^{PPRTV}$ is set by EPA via PPRTV assessments (PPRTV 2026), and $RfC_{i}^{H}$, $RfC_{i}^{L}$, $RfC_{i}^{M}$, $RfC_{i}^{P}$, and $RfC_{i}^{R}$ are the RfCs reported in Hoshino et al. (2025), Lu et al. (2022), Marano et al. (2018), Pack et al. (2018), and Rodrigo et al. (2021), respectively (see the Supporting Information). Similarly to the results reported in Table 1, these choices for the emission data and for parameter values allowed the model in Equation (21) to provide the most comprehensive set of HQ results that can be found in the literature. In Table 2, we can observe that: (a) for both the products, 37 (out of 53) compounds show no potential for adverse noncancer effects ($HQ_{i}<1$); (b) in the case the HTP aerosol, this holds true for 46 compounds. Moreover, the HQ for compounds included in the HTP aerosol is reduced compared to the same compounds in the 3R4F cigarette by a factor between 5.13% and 99.94%, depending on the compound, while for Cobalt the HQ increases.

**TABLE 2:** HQ results for 53 compounds computed using the model in Equation (21) on data for the 3R4F reference cigarette and the THS2.2‐R, and percentage reduction in using the HTP compared to the cigarette.

Compound	CAS	HQ	HQ	HQ
		3R4F	THS2.2	% Red.
2‐Nitropropane	79‐46‐9	6.00e−01	3.80e−04	99.94
Cadmium	7440‐43‐9	5.26e+00	1.11e−02	99.79
Isoprene	78‐79‐5	4.39e−01	1.06e−03	99.76
1,3‐Butadiene	106‐99‐0	4.44e+01	1.36e−01	99.69
Acrylonitrile	107‐13‐1	1.47e+01	8.33e−02	99.43
p‐Cresol	106‐44‐5	2.00e−02	1.44e−04	99.28
Benzene	71‐43‐2	2.97e+01	2.37e−01	99.20
Hydrogen cyanide	74‐90‐8	4.75e+02	4.15e+00	99.13
m‐Cresol	108‐39‐4	8.34e−03	7.30e−05	99.12
Ethylbenzene	100‐41‐4	1.50e−02	1.35e−04	99.10
Ethylene oxide	75‐21‐8	7.58e−01	6.88e−03	99.09
m+p‐Cresol		1.61e−02*	1.70e−04	98.95
Toluene	108‐88‐3	5.29e−01	6.31e−03	98.81
Naphthalene	91‐20‐3	2.65e−01	3.60e−03	98.64
o‐Cresol	95‐48‐7	1.01e−02	1.49e−04	98.52
CO	630‐08‐0	4.48e+00	7.03e−02	98.43
Nitromethane	75‐52‐5	4.50e−01	8.96e−03	98.01
Vinyl chloride	75‐01‐4	4.86e−02	1.07e−03	97.80
Resorcinol	108‐46‐3	1.38e−03	3.92e−05	97.16
NOx		1.18e+00	3.56e−02	96.99
Styrene	100‐42‐5	1.93e−02	6.94e−04	96.41
Beryllium	7440‐41‐7	6.77e−02	2.56e−03	96.21
MEK	78‐93‐3	3.62e−02	1.46e−03	95.96
Acetone	67‐64‐1	2.09e−02	1.12e−03	94.63
m‐Xylene	108‐38‐3	1.94e−01*	1.08e−02*	94.43
Acrolein	107‐02‐8	7.61e+03	4.36e+02	94.27
Benzo[a]pyrene	50‐32‐8	6.99e+00	4.13e−01	94.10
Crotonaldehyde	4170‐30‐3	6.10e+00	4.55e−01	92.54
Hydroquinone	123‐31‐9	9.99e−01	7.81e−02	92.19
Vinyl Acetate	108‐05‐4	3.57e−03	3.16e−04	91.17
Phenol	108‐95‐2	7.18e−02	7.50e−03	89.56
Formaldehyde	50‐00‐0	9.44e+00	1.06e+00	88.78
Propionaldehyde	123‐38‐6	1.50e+01	1.72e+00	88.51
Propylene oxide	75‐56‐9	3.78e−02	4.64e−03	87.73
Arsenic	7440‐38‐2	5.30e−01	6.70e−02	87.36
Acetaldehyde	75‐07‐0	1.82e+02	2.35e+01	87.09
Catechol	120‐80‐9	6.56e−01	1.03e−01	84.26
Lead	7439‐92‐1	8.16e−03	1.51e−03	81.51
Pyridine	110‐86‐1	2.74e−01	5.65e−02	79.42
Hydrazine	302‐01‐2	3.16e−01	6.80e−02	78.52
Selenium	7782‐49‐2	2.04e−04	5.44e−05	73.32
n‐Heptanal	111‐71‐7	7.33e+00*	2.03e+00*	72.27
Butyraldehyde	123‐72‐8	6.76e−01	2.02e−01	70.08
Nitrobenzene	98‐95‐3	7.81e−03	2.80e−03	64.08
Ammonia	7664‐41‐7	1.58e−01	5.97e−02	62.15
Acrylamide	79‐06‐1	7.55e−01	2.90e−01	61.62
Mercury	7439‐97‐6	1.45e−01	5.76e−02	60.29
Nicotine	54‐11‐5	3.31e+02	2.16e+02	34.76
Chromium	7440‐47‐3	4.32e‐01	2.86e−01	33.69
Nickel	7440‐02‐0	1.32e+00	9.99e−01	24.08
Furfural	98‐01‐1	9.05e−01	7.39e−01	18.31
2,3,7,8‐Tetra CDD	1746‐01‐6	9.75e−02*	9.25e−02*	5.13
Cobalt	7440‐48‐4	3.82e−01	4.04e−01	−5.83

*HQ results for compounds whose means come from a single data source.

### 4.2 Margin of Exposure

The MOE of a compound is the ratio between a toxicological reference point (the PoD) and the estimated human exposure level to that compound (EFSA Scientific Committee et al. 2025). MOE expresses how higher the dose that causes no effect in studies is as compared to the actual human exposure. The higher the MOE, the lower the health hazard. Generally, an MOE much greater than 100 is assumed of low concern, as the exposure is far below the level causing adverse effects. On the other side, MOEs lower than 100 indicate potential concerns, as the margin between the no‐effect level and the estimated exposure falls below the default uncertainty factor of 100. Only at an MOE below 1 would the exposure actually exceed the no‐effect level.

MOE results from different models, such as Kusonić et al. (2023) and Rodrigo et al. (2021), are very different and not comparable even when using the same emission values. However, these models can be used to estimate the MOE percentage reduction in using cigarettes compared to HTPs, that is,

(24) $$\begin{array}{c} MOE_{i}\;\%\;Reduction=100*\left(1-\frac{MOE_{i}^{Cig}}{MOE_{i}^{HTP}}\right). \end{array}$$

In Table B.1, we report the MOE results for the THS2.2‐R and the 3R4F reference cigarette computed via the models described in Kusonić et al. (2023) (MOE1) and Rodrigo et al. (2021) (MOE2) using their specific parameter values and the same emission values from the HTP‐AeroChem data set used for results in Tables 1 and 2, as well as the percentage reduction in using the 3R4F cigarette compared to the HTP. Here, it can be observed that, even though the results with the two models strongly differ, the percentage reduction is the same.

**TABLE 3:** MOE results for 53 compounds computed using the model in Equation (25) on data for the 3R4F reference cigarette and the THS2.2‐R, and percentage reduction in using the cigarette compared to the HTP.

Compound	CAS	MOE	MOE	MOE
		3R4F	THS2.2	% Red.
2‐Nitropropane	79‐46‐9	2.04e+00	2.70e+03	99.92
Cadmium	7440‐43‐9	1.90e−01	1.02e+02	99.81
Isoprene	78‐79‐5	2.27e+00	9.55e+02	99.76
1,3‐Butadiene	106‐99‐0	2.26e−02	7.62e+00	99.70
Acrylonitrile	107‐13‐1	6.88e−02	1.24e+01	99.45
Benzene	71‐43‐2	3.34e−02	4.69e+00	99.29
p‐Cresol	106‐44‐5	4.99e+01	6.99e+03	99.29
Hydrogen cyanide	74‐90‐8	2.08e−03	2.56e‐01	99.19
m‐Cresol	108‐39‐4	1.21e+02	1.39e+04	99.13
Ethylene oxide	75‐21‐8	1.29e+00	1.48e+02	99.13
Ethylbenzene	100‐41‐4	6.74e+01	7.40e+03	99.09
m+p‐Cresol		6.20e+01*	6.54e+03	99.05
Naphthalene	91‐20‐3	3.89e+00	3.91e+02	99.00
Toluene	108‐88‐3	1.88e+00	1.57e+02	98.80
o‐Cresol	95‐48‐7	9.88e+01	6.74e+03	98.53
CO	630‐08‐0	2.24e−01	1.42e+01	98.42
Nitromethane	75‐52‐5	2.46e+00	1.16e+02	97.87
Vinyl chloride	75‐01‐4	2.07e+01	9.07e+02	97.72
Resorcinol	108‐46‐3	7.20e+02	2.55e+04	97.17
NOx		8.47e−01	2.84e+01	97.02
Styrene	100‐42‐5	5.04e+01	1.57e+03	96.79
Beryllium	7440‐41‐7	1.19e+01	3.60e+02	96.70
MEK	78‐93‐3	2.76e+01	6.67e+02	95.86
Acetone	67‐64‐1	4.74e+01	8.67e+02	94.53
m‐Xylene	108‐38‐3	5.15e+00*	9.26e+01*	94.43
Benzo[a]pyrene	50‐32‐8	1.44e−01	2.50e+00	94.21
Acrolein	107‐02‐8	1.30e−04	2.22e−03	94.13
Crotonaldehyde	4170‐30‐3	1.63e−01	2.35e+00	93.04
Hydroquinone	123‐31‐9	1.00e+00	1.33e+01	92.46
Vinyl Acetate	108‐05‐4	2.85e+02	3.13e+03	90.88
Phenol	108‐95‐2	1.40e+01	1.50e+02	90.68
Formaldehyde	50‐00‐0	1.07e−01	1.06e+00	89.86
Propionaldehyde	123‐38‐6	6.61e−02	5.80e−01	88.60
Arsenic	7440‐38‐2	1.88e+00	1.56e+01	87.96
Propylene oxide	75‐56‐9	2.59e+01	2.15e+02	87.95
Acetaldehyde	75‐07‐0	5.41e−03	4.38e−02	87.64
Catechol	120‐80‐9	1.52e+00	9.68e+00	84.29
Hydrazine	302‐01‐2	2.95e+00	1.47e+01	79.94
Pyridine	110‐86‐1	3.61e+00	1.76e+01	79.49
Lead	7439‐92‐1	1.24e+02	5.92e+02	79.01
Selenium	7782‐49‐2	4.85e+03	2.20e+04	77.98
n‐Heptanal	111‐71‐7	1.36e−01*	4.92e−01*	72.27
Butyraldehyde	123‐72‐8	1.48e+00	5.12e+00	71.17
Nitrobenzene	98‐95‐3	1.06e+02	3.48e+02	69.51
Ammonia	7664‐41‐7	6.33e+00	1.69e+01	62.43
Acrylamide	79‐06‐1	1.33e+00	3.53e+00	62.38
Mercury	7439‐97‐6	6.86e+00	1.72e+01	60.20
Chromium	7440‐47‐3	2.29e+00	3.89e+00	41.20
Nicotine	54‐11‐5	3.02e−03	4.69e‐03	35.67
Nickel	7440‐02‐0	8.01e−01	1.21e+00	33.99
Furfural	98‐01‐1	1.11e+00	1.33e+00	16.89
2,3,7,8‐Tetra CDD	1746‐01‐6	1.03e+01*	1.08e+01*	5.13
Cobalt	7440‐48‐4	3.09e+00	2.85e+00	−8.59

*MOE results for compounds whose means come from a single data source.

For the purpose of estimating the percentage reduction as in Equation (24), the MOE for compound i can be modeled as the inverse of its HQ

(25) $$\begin{array}{c} MOE_{i}=\frac{1}{HQ_{i}}=10^{3}\frac{RfC_{i}}{EC_{i}}, \end{array}$$

disregarding any uncertainty factors for deriving reference values $RfC_{i}$ from PoDs, as their contribution is eliminated when considering the quotient in Equation (24).

Table 3 compares the MOE results for 53 compounds found in the mainstream smoke of the 3R4F reference cigarette with those for the same compounds found in the aerosol of the THS2.2‐R under the HCI smoking regimen, and provides the MOE percentage reduction in using the cigarette compared to the HTP. Specifically, the emission data and model parameter values are the same as those adopted for the comparisons in Tables 1 and 2, while values for $RfC_{i}^{P}$ in Equation (25) are the most conservative among the available values, that is, the minima in the set defined in Equation (23) (see the Supporting Information). Similarly to the results reported in Tables 1 and 2, these choices for the emission data and for parameter values allowed the model in Equation (25) to provide the most comprehensive set of MOE results that can be found in the literature. In Table 3, we observe a generally strong decrease of MOE when using the 3R4F as compared to the HTP, with percentage reductions between 5.13% and 99.92%. Only for Cobalt a very tiny MOE increase is registered.

## 5 Aggregated Results

Since the CRs and noncancer hazards are related to each specific compound in the smoke/aerosol of a given product, the aggregation of results obtained by all the considered compounds is often assumed as an indication of the risk/hazard of the product itself.

Specifically, for a tobacco product P whose smoke/aerosol contains n cancerogenic compounds, the aggregated lifetime cancer risk $LCR^{P}$ is estimated as

(26) $$\begin{array}{c} LCR^{P}=\sum_{i=1}^{n}LCR_{i}. \end{array}$$

This aggregation, adopted, for example, in Pack et al. (2019) and Rodrigo et al. (2021), is based on the assumption that the carcinogenic effects are additive across all carcinogens. However, this assumption is rarely the case, due to the potential for synergistic or antagonistic interactions among constituents.

Similarly, for a tobacco product P whose smoke/aerosol contains m compounds potentially causing noncancer effects, the aggregated HQ, often called Hazard Index, $HI^{P}$, is estimated as

(27) $$\begin{array}{c} HI^{P}=\sum_{i=1}^{m}HQ_{i}. \end{array}$$

The HI is adopted, for example, in Pack et al. (2018;2019) and Lu et al. (2022). Besides the same assumption on additivity of noncancer effects, in this case, the aggregation should be performed only according to the target organ or effect, as the aggregated compounds should share a common mechanism of toxicity (i.e., the same organ or biochemical pathway) and exhibit similar toxicological endpoints. This point is well addressed, for example, in Pack et al. (2019), where cumulative and organ‐specific aggregated values are compared, showing that the cumulative ones supersede any conclusion provided by the organ‐specific aggregations.

Finally, the aggregated MOE for a product P whose smoke/aerosol contains m compounds potentially causing noncancer effects, $MOE_{T}^{P}$, can be obtained by the reciprocal's rule as

(28) $$\begin{array}{c} MOE_{T}^{P}=\frac{1}{\sum_{i=1}^{m}\frac{1}{MOE_{i}}}, \end{array}$$

sharing with the HI the base assumptions and applicability constraints (EFSA Scientific Committee et al. 2019). It is used as a rough estimate of additive cumulative exposure in Lachenmeier et al. (2018) and Rodrigo et al. (2021), but explicitly disregarding any differences in target site, toxicological mechanism, or potential synergistic interactions of compounds.

**FIGURE 4:**
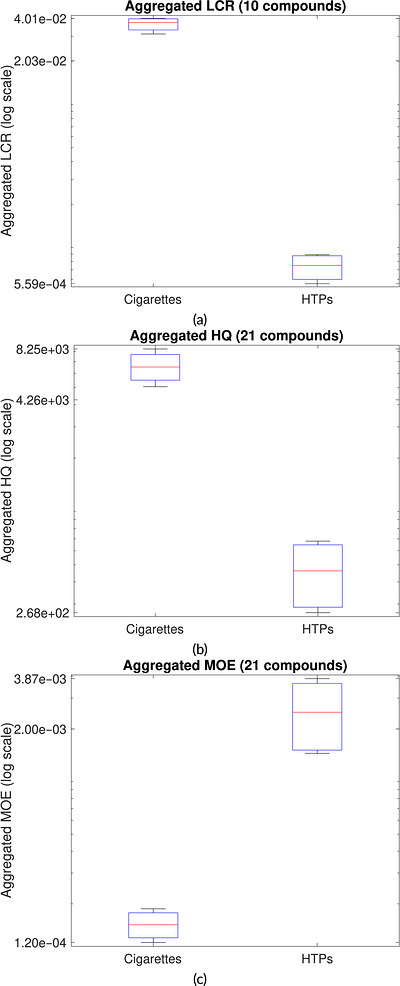
Box plots of (a) $LCR^{P}$ values obtained using Equation (26), (b) $HI^{P}$ values obtained using Equation (27), and (c) $MOE_{T}^{P}$ values obtained using Equation (28), on pooled means from the HTP‐AeroChem data set for various products, grouping them into Cigarettes (3R4F, 1R6F, 1R5F, and CC) and HTPs (THS2.2‐R, THS2.2‐M, THP1.0‐R, and THP1.0‐M).

Examples of aggregated results are shown in Figure 4. Here, the box plots of aggregated values were obtained on pooled means from the HTP‐AeroChem data set for various products, grouping them into Cigarettes (3R4F, 1R6F (University of Kentucky 2024a), 1R5F (University of Kentucky 2024b), and CC) and HTPs (THS2.2‐R, THS2.2‐M, THP1.0‐R, and THP1.0‐M). Model parameters were chosen as follows: $RR_{i}=100$ and MS=0 in Equation (14); NCd = 20 and DBV = 20 in Equation (15); NCy=365∗NCd, LE = 70, and SA = 0 in Equation (16); $IUR_{i}$ in Equation (17) as specified in Equation (20); $RfC_{i}$ in Equation (21) and in Equation (25) as minima in the set specified in Equation (23). Box plots are shown using a logarithmic *y*‐axis. Aggregated LCR values range in [3.12 × 10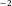, 4.01 × 10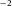] for Cigarettes and in [5.59 × 10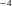, 8.92 × 10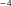] for HTPs. HI values range in [5.06 × 10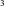, 8.25 × 10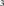] for Cigarettes and in [2.68 × 10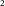, 6.80 × 10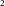] for HTPs. Finally, aggregated MOE values range in [1.20 × 10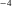, 1.87 × 10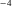] for Cigarettes and in [1.45 × 10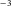, 3.87 × 10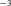] for HTPs.

## 6 Analysis of the Results

Given the versatility of the risk assessment models described in Sections 3 and 4, here we use them in several ways:

(1) to reproduce the results of existing models by applying the same emission data and parameter values reported in the literature, to demonstrate the generality of our formulation;
(2) to vary model parameters while keeping emissions fixed, to illustrate the role of individual parameters;
(3) to analyze the distribution of risk assessment outcomes when multiple emission data sets for the same compounds are available.

### 6.1 Reproducing Existing Models

Results of the CR model of Kusonić et al. (2023) can be reproduced using our LCR model by: (a) using the CC emission values reported in Jaccard et al. (2017); (b) setting RR rates to 100% ($RR_{i}$ = 100) and MS to zero (MS = 0); (c) fixing DBV = 20; (d) varying NCd and assuming NCy = 365*NCd; (e) setting LE = 70 and SA = 0; and (f) using the Slope Factors for the 10 carcinogenic compounds provided in their supplementary material (instead of IURs). For NCd = 20, the results shown in Table B.2 closely match those in the supplementary material of Kusonić et al. (2023). The aggregated value $LCR^{CC}$ is 8.78 $\times 10^{-3}$.

Results of the model by Pack et al. (2018) can be reproduced using our LCR formulation by: (a) using the emission values for 18 compounds in CC smoke reported by the authors; (b) using the DBV, NCd, NCy, LE, and SA values reported by the authors separately for males and females; (c) applying the MS values and compound‐specific RR rates and using the IURs for the 18 carcinogenic compounds listed in their supplementary material. As shown in Table B.3, our results fit the distributions presented in their Figure 1b. The aggregated $LCR^{CC}$ values are 1.9$\times 10^{-3}$ for females and 3.0 $\times 10^{-3}$ for males.

The results of Rodrigo et al. (2021) for the THS2.2 can be replicated using our LCR model by: (a) using the emission values of 21 compounds in the THS2.2 aerosol reported in their supplementary material; (b) setting RR = 100 and MS = 0; (c) fixing DBV = 20 and NCd = 20, with NCy = 365*NCd; (d) assuming LE = 70 and SA = 0; and (e) using the IURs listed in their Table 1. The results in Table B.4 lead to an aggregated LCR of 3.9 $\times 10^{-3}$, in agreement with the values shown in their Figure 1.

The results of the noncancer model by Pack et al. (2018) can be replicated using our HQ model by: (a) using the emission values for 31 compounds in the smoke of CCs provided by the authors; (b) using the MS and the RR rates given in their supplementary material; (c) using values for DBV, NCd, NCy, LE, and SA provided in their supplementary material separately for Males and Females; (e) using the RfCs of the 31 compounds provided in their supplementary material. The results shown in Table B.5 well fit the distributions given in their Table 4. The aggregated result $HI^{CC}$ over the 31 compounds sums to 1105.9 for Females and 1348.3 for Males, with Acrolein giving sharply the highest contribution.

### 6.2 Varying Model Parameters

Here, we fix the pooled mean emission values of the HTP‐AeroChem data set, and vary, one‐by‐one, the LCR model parameters.

**FIGURE 5:**
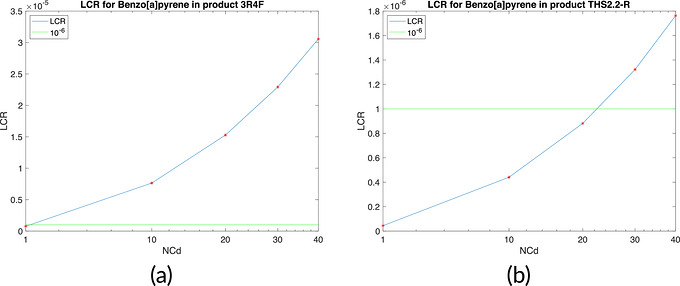
LCR results for Benzo[a]pyrene varying NCd from 1 to 40 for (a) the 3R4F cigarette and (b) the THS2.2‐R HTP.

For example, varying NCd from 1 to 40, LCR results for Benzo[a]pyrene are plotted in Figure 5 for the 3R4F reference cigarette and the THS2.2‐R. Values for $IUR_{i}$ in Equation (17) are set as in Equation (20), while the remaining model parameters were set as $RR_{i}$ = 100, MS = 0, DBV = 20, NCy = 365*NCd, LE = 70, SA = 0. In Figure 5, we can observe that in the smoke of the 3R4F cigarette, the CR of the compound is acceptable (i.e., smaller than 10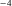), but not negligible (i.e., higher than 10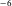), regardless of the NCd. In contrast, for the HTP aerosol, it is negligible when at most 20 sticks are smoked per day, and acceptable otherwise.

**FIGURE 6:**
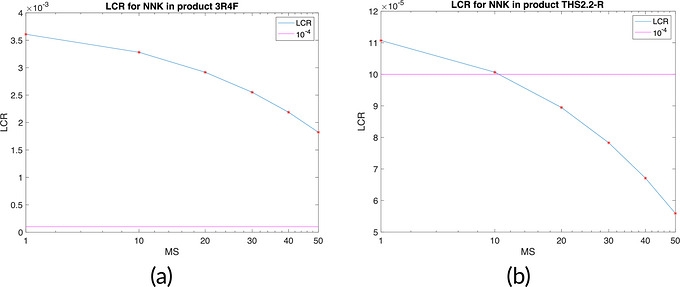
LCR results for NNK varying MS from 1% to 50% for (a) the 3R4F cigarette and (b) the THS2.2‐R HTP.

As another example, varying MS from 1% to 50%, LCR results for NNK are plotted in Figure 6 for the 3R4F reference cigarette and the THS2.2‐R HTP. The remaining parameters are fixed as in the previous example, setting NCd to 20. In Figure 6, we can observe that in the smoke of the 3R4F cigarette the CR of the compound is unacceptable ($>10^{-4}$) whichever is the MS, while in the HTP aerosol it becomes acceptable with an MS greater than 10%.

### 6.3 Varying the Emission Data

**FIGURE 7:**
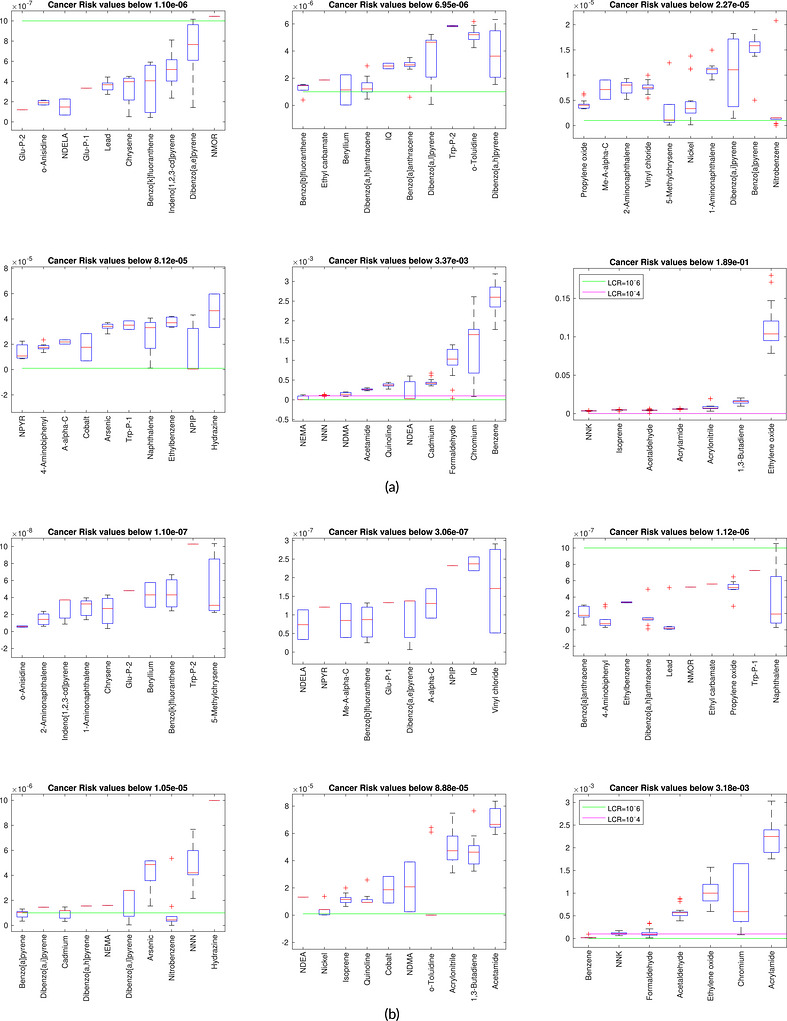
Box plots of LCR results computed using the model in Equation (17) on data from up to 28 different sets of emission values included the HTP‐AeroChem data set for (a) the 3R4F cigarette and (b) the THS2.2‐R HTP.

**FIGURE B.1:**
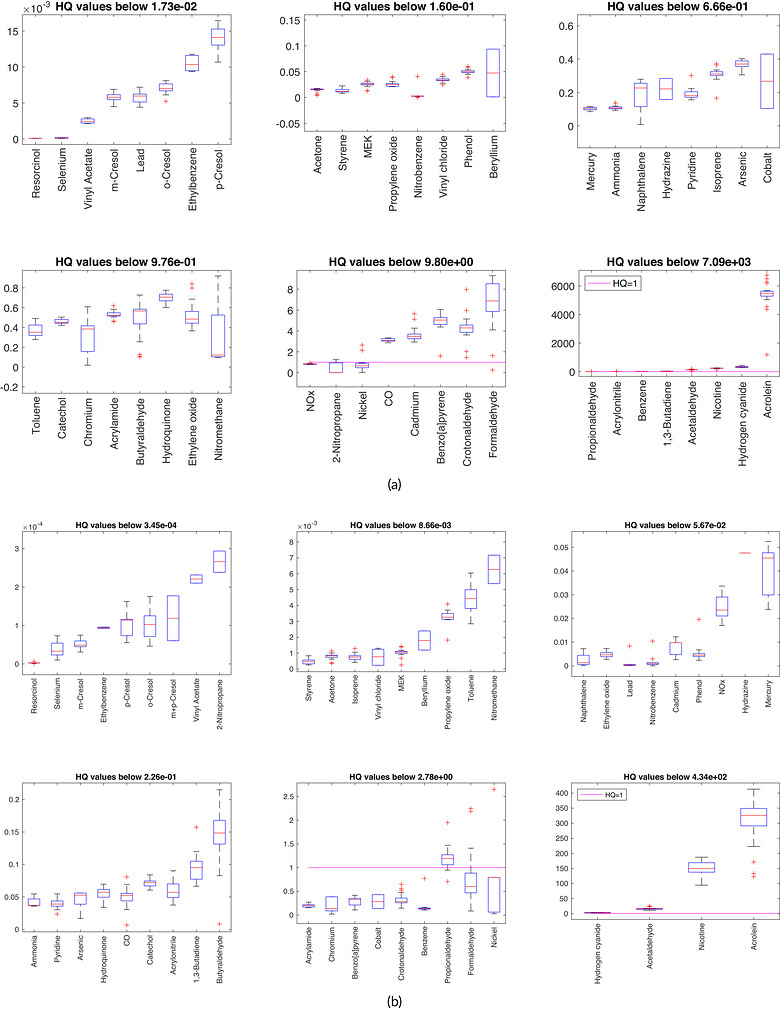
Box plots of HQ results computed using the model in Equation (21) on data from up to 28 different sets of emission values included the HTP‐AeroChem data set for (a) the 3R4F cigarette and (b) the THS2.2‐R HTP.

In these experiments, we use the pooled means of measures included in the HTP‐AeroChem data set, coming from up to 28 different sources for the 3R4F reference cigarette and the THS2.2‐R HTP, and fix the model parameters by setting $RR_{i}$ = 100 and MS = 0 in Equation (14), NCd = 20 and DBV = 20 in Equation (15), NCy = 365*NCd, LE = 70, and SA = 0 in Equation (16), and using as IURs of the carcinogenic compounds in Equation (17) as set in Equation (20). Figure 7 shows box plots of the LCR results for 57 compounds included in the emissions of the two products. Each box plot is related to a variable number (from 2 up to 24) of values for each compound measured in the mainstream aerosol/smoke by one of the different sources. The compounds are shown sorted in increasing order of maximum LCR value for visualization purposes. The green and magenta horizontal lines indicate the thresholds generally adopted for highlighting LCR values considered negligible ($<10^{-6}$), acceptable (in [$10^{-6},10^{-4}$]), or unacceptable ($>10^{-4}$). Analogous box plots for HQ results are provided in Figure B.1. These are obtained using the same emission data and model parameters, and choosing as RfCs of the compounds in Equation (21) the minima in the set given in Equation (23).

## 7 Discussion

The preclinical risk analysis models developed in this study enable a systematic and reproducible comparison of cancer and noncancer risks associated with HTPs versus conventional cigarettes. By formalizing cancer risk (LCR), noncancer hazard (HQ), and MOE models within a unified mathematical structure, we provide a transparent way to incorporate heterogeneous toxicological reference values, machine‐generated emissions data, and user‐behavior assumptions. This supports the regulatory expectation that computational toxicology should serve as a bridge between chemical characterization and in vitro/in vivo toxicological evaluations. Clearly, subsequent clinical and population studies remain fundamental to assess how a novel tobacco product actually impacts human exposure and disease risk.

A key contribution of this work is the demonstration that most models currently used in the literature can be expressed as specific cases of the general equations presented in Sections 3 and 4. This reveals that apparent discrepancies in published risk estimates often stem not from scientific disagreement but from differences in modeling assumptions—such as RR rates, MS, breathing volume, toxicological thresholds, or exposure duration. By varying these parameters one‐at‐a‐time, we showed how individual assumptions can strongly influence model outcomes, highlighting the importance of explicitly reporting parameter choices when communicating risk estimates. Analysis of up to 28 independent emission data sets confirms that variability in machine‐generated yields represents a major source of uncertainty in risk assessment. Box‐plot analyses of LCR values across compounds demonstrate that while absolute risk levels fluctuate significantly across data sets, the qualitative trends—namely, lower LCR values for HTP aerosols compared to cigarette smoke—remain stable. Likewise, HQ and MOE results consistently favor HTPs across multiple modeling approaches, even when models differ substantially in structure, thresholds, or toxicological endpoints. These findings reinforce the idea that computational toxicology provides a robust, model‐agnostic platform for evaluating relative risk, provided that results are interpreted in terms of reduction percentages rather than absolute numbers. Nonetheless, the models presented in Sections 3 and 4 can be adopted to obtain absolute risk assessment estimates for single products, provided that model parameters are available, as we did in Section 6.1 (Tables B.2–B.5). This would be the only alternative in case of chemical compounds whose emission value is known for just a single product (either because the compound is unique to that product or because it has not been measured for the compared product).

The aggregated risk metrics presented in Section 5 serve as an intuitive summary of cancer and noncancer hazards. Nonetheless, they rely on an assumption of additivity that may not hold for all compound classes, and they do not capture potential synergistic or antagonistic interactions among constituents. For example, nicotine is generally not classified as a primary carcinogen by the IARC, and CR from tobacco products is largely attributed to combustion‐ or aerosol‐derived toxicants such as tobacco‐specific nitrosamines, aldehydes, volatile organic compounds (VOCs), and metals. Nevertheless, growing experimental evidence suggests that nicotine can act as a cocarcinogen or tumor promoter, enhancing cancer‐related processes including cell proliferation, angiogenesis, inhibition of apoptosis, invasion, and metastasis (Sanner and Grimsrud 2015). In other words, while nicotine may not directly initiate carcinogenesis, it may amplify the harmful effects of other carcinogens present in cigarette smoke or HTP emissions (Mishra et al. 2015). As a result, risk models based solely on additive toxicant burden may fail to capture important biological interactions and therefore underestimate long‐term disease risk. For this reason, future refinements should consider organ‐specific aggregation, mechanistic clustering of toxicants, or probabilistic mixture‐toxicity frameworks. More advanced modeling, for example, integration of PBPK models or MOA evidence, could further strengthen computational toxicology assessments and improve biological relevance, as recommended by the FDA guidance and recent EFSA reviews.

Overall, our results support the use of quantitative computational toxicology as an essential component of the MRTP assessment paradigm. The flexibility of the proposed framework enables harmonized comparison of products, reproducibility of published models, and sensitivity analyses that clarify the influence of key assumptions—features that are often missing in current risk‐assessment literature.

## 8 Conclusions

This study presents a comprehensive and transparent computational toxicology framework for evaluating cancer and noncancer risks associated with HTPs. By systematically integrating machine‐generated emissions data, toxicological reference values, and user‐exposure assumptions, we reproduced the results of several published models and conducted extensive sensitivity analyses to examine the impact of parameter variability. The general mathematical formulation proposed here unifies disparate approaches found in the literature and highlights the importance of reporting model parameters, exposure assumptions, and toxicological thresholds to ensure reproducibility. Our results emphasize that computational toxicology plays a critical role in bridging chemical analysis and biological evidence. This preclinical process provides a quantitative foundation for assessing product risk prior to clinical and population studies.

Future work will focus on several aspects. First, the incorporation of PBPK and MOA‐based data for improved biological relevance; then, we will further expand and update the database of RR rates, toxicological thresholds, and emission measurements; moreover, we will develop mixture‐toxicity models and organ‐specific aggregation procedures; and finally, we will implement probabilistic frameworks to capture uncertainty across exposure scenarios.

## Author Contributions

Design of the work: L.M., M.R.G.; Acquisition, integration: L.M., B.Y.; Exploratory data analysis, statistical analysis, and interpretation of results: L.M., M.R.G.; Disambiguation of chemical compounds, measurements interoperability and interpretation: F.D.V.B.; Models implementation: B.Y., L.M.; Work draft: L.M., F.D.V.B., M.R.G.; Substantial revision of the work: L.M., F.D.V.B., M.R.G.; Supervision: L.M., M.R.G.

## Conflicts of Interest

The authors declare no conflicts of interest.

## Supporting information

Data S1

## Appendix A Other Models

Baumung et al. (2016) conducted a health risk assessment of nicotine for smokers of cigarettes using the margin of exposure (MOE) approach and compared the results to the literature MOEs of various other tobacco toxicants. The MOE is defined as the ratio of the toxicological threshold (the *benchmark dose*) to the estimated human intake. Dose–response modeling of human and animal data were used to derive the benchmark dose. The MOE was calculated using probabilistic Monte Carlo simulations for daily cigarette smokers.

Esposito et al. (2022) used an MOE approach for neurotoxic and carcinogenic risks, as well as an ILCR approach, following Pack et al. (2018), to estimate the risk associated with exposure to acrylamide present in both heated tobacco product (HTP) aerosol and cigarette smoke.

The daily exposure E to acrylamide (ng/kg BodyWeight/day) is computed as E=C∗NCd/BW, where C is the concentration found in samples (ng/stick), NCd is the number of cigarettes consumed each day (cig/day), and the BodyWeight BW is assumed as 62 and 78 kg for females and males, respectively. Then, the MOE is computed as $MOE=BMDL_{10}/E$, where $BMDL_{10}$ is the benchmark dose lower confidence limit for a 10% response (mg/kg BodyWeight/day), with values chosen based on murine models.

The ILCR of the acrylamide carcinogen is computed as in Equations (6)–(7) by Pack et al. (2018), i carcinogen being acrylamide.

Hirn et al. (2020) used a Quantitative Risk Assessment (QRA) approach to compare noncancer and cancer risk (CR) estimates for emissions generated by a hybrid HTP developed by Japan Tobacco International with smoke from the 3R4F reference cigarette using the ISO and the Health Canada Intense (HCI) smoking regimens.

The Exposure Concentration (EC, in µg/m^3^), based on the analyte concentration in emission time‐weighted to account for the duration of exposure and the activity patterns for smoking/vaping, is estimated as EC=CA∗ET∗EF∗ED/AT, where CA (µg/m^3^) is the analyte Concentration in Air, estimated based on the level of the substance measured in specified puff count (per collection, i.e., 85 puffs for the HTP and 7.8–8.7 puffs for the 3R4F under the ISO regime, and 70 puffs for the HTP or 9.6–11.6 puffs for the 3R4F under the HCI regime) and the volume of air exchanged for 400 puffs; ET (hours/day) is the Exposure Time, computed based on the puffing regimen used for measuring the analytes (i.e., ISO and HCI [2 s duration]) per collection adjusted to 400 puff counts; EF (days/year) is the Exposure Frequency, assumed to be daily (i.e., 365 days/year); ED (years) is the Exposure Duration, set to 54 years based an assumed initiation at 18 years of age and a global life expectancy of 72 years; and AT (ED in years * 365 days/year *24 h/day) is the Averaging Time. Noncancer risks were quantified with the hazard quotient (HQ), computed as HQ=EC/TRV, where TRV (Toxicity Reference Value, µg/m^3^) is extracted according to the EPA tiered approach (for noncancer effects, based on EPA RfCs, CalEPA chronic RELs, or TCEQ long‐term ESLs).

CRs were calculated for the analytes that are listed by FDA as carcinogen on the established list of HPHCs and on the subsequent list of proposed additions to the established list of HPHCs in tobacco products and tobacco smoke. Excess Lifetime Cancer Risks (ELCRs) in a population of 1000 were calculated according to ELCR=IUR∗EC∗1000, with IUR ((µg/m^3^)^−1^) coming from EPA, CalEPA, or TCEQ.

Cumulative noncancer health risks were assessed within five different health outcome domains (addictive compound, cardiovascular toxicant, respiratory toxicant, reproductive or developmental toxicant, and carcinogen) using the HI defined in Equation (27), while the cumulative cancer risk was calculated by determining the incremental increase in the probability of an individual developing cancer over a lifetime, as the sum of the single ELCRs.

Provided data are only those for chemical characterization. The percent reduction of HQs for HTPs against 3R4F for individual analytes are only plotted (in their Figures 2 and 3) and cumulative noncancer risks (HIs) are summarized by health outcome domain in the plot of their Figure 4. However, all remaining parameter values (human health TRVs, individual and cumulative noncancer and CR values) have not been published.

Hoshino et al. (2025) determined the concentrations of flavors, Volatile Organic Compounds (VOCs), tobacco markers, and tobacco‐specific nitrosamines in the mainstream aerosols of HTPs, to assess the risk associated with their use. They also investigated the effects of the heating temperature on the components of the mainstream aerosol, assessed their noncarcinogenic and carcinogenic risks, and evaluated their potential influence on indoor air pollution.

The noncarcinogenic and carcinogenic risks of HTP use were assessed using the HQ or MOE, and CR, respectively, computed as follows.

The HQ value was determined to evaluate the potential health hazards due to the exposure to a contaminant i as $HQ_{i}$ = $\left(I_{i}*ED*10^{-3}\right)/\left(RfC_{i}*A*L\right)$, where $I_{i}$ is the estimated daily Intake of compound i (in µg/day), given by $I_{i}$ = $NCd*C_{i}$, with NCd average number of cigarettes smoked per day, in the experiments set to 15.5, and $C_{i}$ (in µg/item) concentration of compound i in the mainstream smoke/aerosol; ED is the number of years of smoking, in the experiments set to 64; $RfC_{i}$ (in mg/m^3^) is the reference concentration of compound i; A (in m^3^/day) is the daily inhalation volume, in the experiments set to 16.5 m^3^/day; L is the expected lifespan, in the experiments set to 84 years.

For compounds with unknown RfC, instead of HQ they computed the MOE to evaluate the potential for noncancer risk, according to $MOE_{i}=\left(NOAEC_{i}*A*L\right)/\left(I_{i}*ED*10^{-3}\right)$, where $NOAEC_{i}$ (in mg/m^3^) is No Observed Adverse Effect Concentration, that is, the concentration of compound i that did not cause any observed adverse effects. The threshold for MOE was set to 100.

Finally, CR was computed as $CR_{i}=\left(I_{i}*IUR_{i}*ED\right)/\left(A*L\right)$, where $IUR_{i}$ (in ${\left(\mu g/m^{3}\right)}^{-1}$) is the Inhalation Unit Risk (IUR), that is, the lifetime Cancer Risk from continuous inhalation exposure to 1 µg of the carcinogen per m^3^ of air. The threshold for CR was set to 10^−6^.

Available model parameter values include RfC, IUR, and NOAEC for 20 compounds (their Table S6). Available data used in the experiments are related to three HTPs with 26 different items, under both ISO and HCI regimens. Data include the concentrations $C_{i}$ of 70 compounds in the mainstream of the HTPs for each of the three replicates (their Table S7). HQ, MOE, and CR results for each product and each replicate are also provided (their tables S10–S12).

Lachenmeier and Rehm (2015) presented a comparative risk assessment of drugs, including alcohol and tobacco, using the MOE approach. Median lethal dose values from animal experiments were used to derive the *benchmark dose* (MOE numerator). The human intake (MOE denominator) was calculated for individual scenarios and population‐based scenarios. The MOE was calculated using probabilistic Monte Carlo simulations. Later, Lachenmeier et al. (2018) provided a comparative QRA of tobacco smoke constituents based on the MOE approach, comparing HTPs to conventional tobacco products. The selection of compounds was restricted to those with an MOE below 10,000, the usual threshold for genotoxic carcinogens, according to Baumung et al. (2016). The exposures for smoking 20 cigarettes per day were estimated using probabilistic Monte Carlo simulation based on average and standard deviations reported in three studies comparing HTPs with conventional cigarettes (Schaller et al. 2016; Auer et al. 2017; Jaccard et al. 2017), mixing yield data acquired under both ISO and HCI smoking regimens. They concluded that HTPs reduced the risk of exposure to 9 out of the 20 most toxic compounds beyond an MOE threshold of 10,000. Based on the toxicological thresholds and related endpoints provided by Baumung et al. (2016), the authors computed the average MOE for tobacco constituents by comparing exposure from HTP emissions with that from conventional tobacco smoke (reported in their Table 1). The combined MOE (MOE_*T*_) excluding nicotine is shown as a box plot (their Figure 1).

Lu et al. (2022) compared the emissions of VOCs, carbon monoxide, nicotine, and tar from HTPs, ECs, and Chinese conventional cigarettes, and evaluated their health risks by applying the same smoking regimens (HCI and CRM81 (CORESTA‐CRM81 2015)) and the Mouth‐Spill (MS) loss mechanism. They estimated the noncarcinogenic risk HI of the three types of tobacco products and the LCR. To this aim, for each constituent i in the aerosols of different types of tobacco products, they modeled the systemic smoker uptake $U_{i}$ (mg/puff) as $U_{i}=C_{i}*RR_{i}*\left(1-MS\right)$, according to Pack et al. (2019), where $C_{i}$ is the concentration of the constituent in the mainstream smoke, $RR_{i}$ is the Respiratory Retention rate (%), and MS is the Mouth‐Spill, estimated as 30% according to St. Charles et al. (2013). Then, the exposure dose $CDI_{i}$ of constituent i emitted by different types of tobacco products (mg/(kg‐day)) was modeled as $CDI_{i}=\left(U_{i}*IR*EF*ED\right)/\left(365*BW*AT\right)$, where IR is the intake rate of cigarettes (puff/day), EF is the exposure frequency during an entire year (365 days/year), ED is the exposure duration for smoking (54 years, obtained as average lifetime minus 18 years), BW is the human body weight (62.7 kg), and AT is the time period over which the dose is averaged (noncarcinogenic AT = ED, carcinogenic AT = lifetime), assuming an average lifetime of 72 years.

The noncarcinogenic hazard index $HI_{i}$ of constituent i was computed as $HI_{i}=CDI_{i}/RFD_{i}$, where $RFD_{i}$ is the noncarcinogenic reference dose for toxic pollutants (mg/kg‐day). A value of $HI_{i}$ less than 1 indicates an acceptable health risk, while values of 1 or greater are hazardous. The overall noncarcinogenic risk HI for a given tobacco product was computed as the sum of the $HI_{i}$ values for all constituents in its aerosol.

The Lifetime Cancer Risk $LCR_{i}$ of constituent i was computed as $LCR_{i}=CDI_{i}*SF_{i}$, where $SF_{i}$ is the cancer Slope Factor for constituent i (mg/kg‐day). Although not said explicitly (but deducible from their Figure 6), the overall carcinogenic risk LCR for a given tobacco product is computed as the sum of the $LCR_{i}$ values for all constituents in its aerosol.

Available data include the parameters for risk assessment ($RFD_{i}$ and $SF_{i}$, but also $RFC_{i}$, $IUR_{i}$, mmHg, Vapor pressure (Pa), and $RR_{i}$, in their Table S8), and the value of the uptake $U_{i}$ and of the exposure dose $CDI_{i}$ for different constituents, emitted by HTPs, electronic cigarettes (ECs), and cigarettes.

Marano et al. (2018) focused on the incorporation of QRA into the process for determining substantial equivalence of two products (exemplified as two tobacco products) as an approach to address the question of whether the use of the new product in the same manner as the existing similar product would potentially raise different questions of public health. In the absence of long‐term epidemiological studies, QRA is a practical and efficient approach for evaluating potential human health risks associated with tobacco products, particularly useful for a relative or comparative assessment of two or more tobacco products. Risk assessment is described as a four‐step process, including hazard identification, exposure assessment, toxicity assessment, and risk characterization. The paper describes how to implement all these steps, providing equations for computing the Exposure Concentration, the Chronic Daily Intake, the ELCR, and the HQ, and detailing all the involved parameters and their choices in accordance with US FDA regulations.

Available data include inhalation toxicity values (RfC and IUR) for TobReg‐18 HPHCs (their Table 6) and values for the exposure parameters adopted in their equations (their Table 8). Instead, the comparison examples reported in the paper are generated using randomly generated numbers; they also make an Excel file implementing their formulas available, to be used with real yield values.

Pack et al. (2019) performed a risk assessment of toxicants on 38 compounds included in the WHO TobReg priority list using human‐smoked yields of Korean smokers, rather than machine‐smoked yields according to conventional regimens, for five different commercial Korean cigarettes. The human‐smoked toxicant yields were estimated from spent cigarette butts and converted to systemic uptake, as in Lu et al. (2022). The ILCR, cumulative ILCR, HQ, HI, and MOE were estimated from the systemic uptake of toxicants combined with Korean exposure factors by gender and age group, as well as for total smokers, similarly to Pack et al. (2018).

Besides data for chemical characterization, available data for risk assessment include statistics for the adopted exposure factors in Korean smokers (their Table 2), barplots for ILCR of 15 carcinogenic toxicants and for HQ of 26 noncarcinogenic toxicants (their Figures 1–2), MOE values of 37 toxicants (their Table 4), and comparisons of cancer and noncancer risks of individual toxicants with previous risk assessments (their Tables 5–6).

Slob et al. (2020) compared the harmful health effects related to two different tobacco products by focusing on the change in cumulative exposure (CCE) of the compounds they emit. The method consists of six steps. The first three steps encompass dose–response analysis of cancer data, resulting in relative potency factors (RPF, estimated from animal data) with confidence intervals. The fourth step evaluates the emission data, yielding confidence intervals for the expected emissions of each compound. The fifth step calculates the change in CCE, probabilistically, resulting in an uncertainty range for the CCE. The sixth step estimates the associated health impact by combining the CCE with relevant dose–response information. The method is applied to eight carcinogens that occur in both HTP emissions and tobacco smoke. Following Rodrigo et al. (2021), the method is limited to the availability of carcinogenicity study data to estimate RPF corresponding to the considered compounds. In Staal, Bil, et al. (2022), the authors extended their method by adding four more compounds for carcinogenicity and five or seven (depending on the chosen PoD) compounds for noncancer cases for predicting changes in the cumulative exposure of HTPs. They found two adverse effects for noncancer diseases associated with tobacco emissions, but still for a very low number of compounds.

Available data include lower and upper bounds for the RPFs of the eight compounds for HTPs and cigarettes based on data from Schaller et al. (2016); Forster et al. (2018) (their Table III), to be combined with the related concentrations to compute the CCE.

Staal,Bos, et al. (2022) summarized methods that can be used to assess the health risks associated with the use of Tobacco and Related Products (TRPs) to: (1) identify the emission components of highest health concern; (2) determine the absolute risk of a TRP; or (3) determine the relative risk of a TRP compared to a reference product (e.g., cigarette). Methods are grouped as Evaluation Frameworks, methods based on individual components (TTC, HQ/HI, MOE, RPF), or methods for the product as a whole (using epidemiological data, in vivo studies, or in vitro models).

Stephens (2018) quantified relative harm caused by inhaling the aerosol emissions of ECs compared with tobacco cigarettes, the THS HTP, and a nicotine inhaler. The cancer potencies of various EC aerosols were modeled using published chemical analyses of emissions and their associated IURs. Potencies were compared using a conversion procedure for expressing smoke and EC vapors in common units. Lifetime cancer risk was calculated from potencies using daily consumption estimates.

Available data include $IUR_{i}$ ((µg/m^3^)^−1^) and average concentrations (µg/mL) for IARC type 1 and 2 carcinogens measured in tobacco smoke and HTP/EC aerosol using data by Schaller et al. (2016), Counts et al. (2005), Pazo et al. (2016), Bodnar et al. (2012), Uchiyama et al. (2018), and various other papers (their Table 1).

## Appendix B Further Tables and Figures

**TABLE B.1:** MOE results for 22 compounds computed using the models described in Kusonić et al. (2023) (MOE1) and Rodrigo et al. (2021) (MOE2) on data for the THS2.2‐R and the 3R4F reference cigarette, and percentage reduction in using the cigarette compared to the HTP.

Compound	CAS	MOE1	MOE1	MOE1	MOE2	MOE2	MOE2
		THS2.2‐R	3R4F	% Red.	THS2.2‐R	3R4F	% Red.
Acetaldehyde	75‐07‐0	3.04e+03	3.76e+02	87.64	4.38e−02	5.41e−03	87.64
Acetone	67‐64‐1	1.30e+05	7.11e+03	94.53	5.78e+03	3.16e−02	94.53
Acrolein	107‐02‐8	2.77e+01	1.63e+00	94.13	2.22e‐03	1.30e−04	94.13
Acrylonitrile	107‐13‐1	3.11e+03	1.72e+01	99.45	1.24e+01	6.88e−02	99.45
Benzene	71‐43‐2	9.39e+03	6.68e+01	99.29	4.69e+00	3.34e−02	99.29
Benzo[a]pyrene	50‐32‐8	5.74e+05	3.32e+04	94.21	2.50e+00	1.44e−01	94.21
Cadmium	7440‐43‐9	2.54e+04	4.75e+01	99.81	1.02e+02	1.90e−01	99.81
Catechol	120‐80‐9	1.14e+04	1.79e+03	84.29	2.07e+01	3.26e+00	84.29
Formaldehyde	50‐00‐0	1.13e+04	1.15e+03	89.86	1.36e+00	1.38e−01	89.86
Hydrogen cyanide	74‐90‐8	3.04e+03	2.47e+01	99.19	2.88e+00	2.34e−02	99.19
Hydroquinone	123‐31‐9	1.89e+04	1.43e+03	92.46	1.67e+02	1.25e+01	92.46
Isoprene	78‐79‐5	1.41e+05	3.35e+02	99.76	4.01e+03	9.54e+00	99.76
MEK	78‐93‐3	3.96e+05	1.64e+04	95.86	6.67e+02	2.76e+01	95.86
Mercury	7439‐97‐6	5.75e+03	2.29e+03	60.20	1.72e+01	6.86e+00	60.20
Nicotine	54‐11‐5	5.24e+00	3.37e+00	35.67	4.69e‐03	3.02e‐03	35.67
Phenol	108‐95‐2	4.70e+05	4.38e+04	90.68	1.50e+02	1.40e+01	90.68
Pyridine	110‐86‐1	7.34e+02	1.50e+02	79.49	8.80e+01	1.81e+01	79.49
Resorcinol	108‐46‐3	2.50e+06	7.07e+04	97.17	2.55e+04	7.20e+02	97.17
Styrene	100‐42‐5	1.75e+06	5.60e+04	96.79	1.57e+03	5.04e+01	96.79
Toluene	108‐88‐3	6.22e+05	7.44e+03	98.80	1.57e+02	1.88e+00	98.80
m+p‐Cresol		1.00e+07	9.50e+04	99.05	6.54e+03	6.20e+01	99.05
o‐Cresol	95‐48‐7	3.92e+06	5.74e+04	98.53	1.18e+04	1.72e+02	98.53

**TABLE B.2:** LCR results for 10 compounds replicating those of Kusonić et al. (2023) for CCs.

Compound	CAS	LCR
Acetaldehyde	75‐07‐0	2.94e−03
1,3‐Butadiene	106‐99‐0	2.36e‐03
Acrylonitrile	107‐13‐1	1.42e−03
Formaldehyde	50‐00‐0	1.34e−03
Benzene	71‐43‐2	5.34e−04
Cadmium	7440‐43‐9	9.40e−05
NNN	16,543‐55‐8	4.05e−05
NNK	64,091‐91‐4	3.05e−05
Benzo[a]pyrene	50‐32‐8	1.50e−05
Lead	7439‐92‐1	3.78e−07

**TABLE B.3:** LCR results for 18 compounds replicating those of Pack et al. (2018) for CCs using parameter values for females (F) and males (M).

Compound	CAS	LCR (F)	LCR (M)
1,3‐Butadiene	106‐99‐0	5.31e−04	8.39e−04
Acetaldehyde	75‐07‐0	5.16e−04	8.14e−04
Acrylonitrile	107‐13‐1	1.88e−04	2.96e−04
Quinoline	91‐22‐5	1.73e−04	2.73e−04
Formaldehyde	50‐00‐0	1.47e−04	2.33e−04
Chromium	7440‐47‐3	1.28e−04	2.01e−04
Benzene	71‐43‐2	1.27e−04	2.01e−04
NNK	64,091‐91‐4	5.21e−05	8.22e−05
4‐Aminobiphenyl	92‐67‐1	5.07e−06	8.00e−06
Arsenic	7440‐38‐2	4.74e−06	7.48e−06
Cadmium	7440‐43‐9	3.60e−06	5.68e−06
Isoprene	78‐79‐5	2.16e−06	3.40e−06
1‐Aminonaphthalene	134‐32‐7	2.14e−06	3.37e−06
Nickel	7440‐02‐0	1.22e−06	1.92e−06
Benzo[a]pyrene	50‐32‐8	8.06e−07	1.27e−06
2‐Aminonaphthalene	91‐59‐8	7.19e−07	1.13e−06
NNN	16,543‐55‐8	5.21e−07	8.22e−07
Lead	7439‐92‐1	1.34e−07	2.11e−07

**TABLE B.4:** LCR results for 21 compounds replicating those of Rodrigo et al. (2021) for the THS2.2‐R HTP.

Compound	CAS	LCR
Acrylamide	79‐06‐1	2.37e−03
Ethylene oxide	75‐21‐8	6.87e−04
Acetaldehyde	75‐07‐0	5.84e−04
Formaldehyde	50‐00‐0	1.04e−04
Acetamide	60‐35‐5	6.67e−05
1,3‐Butadiene	106‐99‐0	5.81e−05
Acrylonitrile	107‐13‐1	4.57e−05
Benzene	71‐43‐2	1.58e−05
Arsenic	7440‐38‐2	5.17e−06
Nickel	7440‐02‐0	4.14e−06
NNN	16,543‐55‐8	4.09e−06
Cadmium	7440‐43‐9	1.18e−06
Benzo[a]pyrene	50‐32‐8	1.03e−06
Propylene oxide	75‐56‐9	5.03e−07
Benzo[a]anthracene	56‐55‐3	2.29e−07
Vinyl chloride	75‐01‐4	1.71e−07
Dibenzo[a,h]anthracene	53‐70‐3	1.49e−07
4‐Aminobiphenyl	92‐67‐1	5.74e−08
o‐Toluidine	95‐53‐4	4.61e−08
Lead	7439‐92‐1	1.94e−08
2‐Aminonaphthalene	91‐59‐8	1.41e−08

**TABLE B.5:** HQ results for 31 compounds replicating those of Pack et al. (2018) for CCs using parameter values for females (F) and males (M).

Compound	CAS	HQ (F)	HQ (M)
Acrolein	107‐02‐8	9.82e+02	1.20e+03
Acetaldehyde	75‐07‐0	4.94e+01	6.03e+01
Hydrogen cyanide	74‐90‐8	4.06e+01	4.95e+01
1,3‐Butadiene	106‐99‐0	1.68e+01	2.05e+01
Propionaldehyde	123‐38‐6	4.57e+00	5.57e+00
Acrylonitrile	107‐13‐1	2.62e+00	3.20e+00
Formaldehyde	50‐00‐0	2.39e+00	2.92e+00
Crotonaldehyde	4170‐30‐3	1.57e+00	1.91e+00
Benzo[a]pyrene	50‐32‐8	1.28e+00	1.55e+00
Benzene	71‐43‐2	1.03e+00	1.26e+00
Chromium	7440‐47‐3	1.01e+00	1.23e+00
Nickel	7440‐02‐0	6.35e−01	7.74e−01
Catechol	120‐80‐9	5.88e−01	7.17e−01
NOx		4.33e−01	5.28e−01
Butyraldehyde	123‐72‐8	3.00e−01	3.66e−01
Hydroquinone	123‐31‐9	2.78e−01	3.38e−01
Cadmium	7440‐43‐9	1.90e−01	2.31e−01
Arsenic	7440‐38‐2	1.39e−01	1.70e−01
Phenol	108‐95‐2	5.63e−02	6.86e−02
Pyridine	110‐86‐1	4.24e−02	5.17e−02
Ammonia	7664‐41‐7	2.61e−02	3.18e−02
Cresol mixture		1.16e−02	1.41e−02
Toluene	108‐88‐3	1.16e−02	1.41e−02
Mercury	7439‐97‐6	9.65e−03	1.18e−02
MEK	78‐93‐3	9.62e−03	1.17e−02
Lead	7439‐92‐1	5.88e−03	7.17e−03
Acetone	67‐64‐1	5.65e−03	6.89e−03
Styrene	100‐42‐5	3.84e−03	4.68e−03
CO	630‐08‐0	4.45e−04	5.42e−04
Resorcinol	108‐46‐3	3.51e−04	4.28e−04
Selenium	7782‐49‐2	1.36e−04	1.65e−04
